# A systematic review protocol for slow-paced breathing in healthy populations: Impacts on cognition and insights into mechanisms of action

**DOI:** 10.1186/s13643-025-03004-w

**Published:** 2025-12-07

**Authors:** Sadhna D. Ramquar, Chayanika Tyagi, Bhanu Sharma, Cynthia Chui, Alexandra Wasti, Robin E. Green

**Affiliations:** 1 Rehabilitation Sciences Institute, University of Toronto, 500 University Avenue, Toronto, ON M5G 1V7 Canada; 2 KITE — Toronto Rehabilitation Institute, University Health Network, 550 University Avenue, Toronto, ON M5G 2A2 Canada; 3 McMaster University, 1280 Main Street West, Hamilton, ON L8S 4L8 Canada; 4 Library and Information Services, Toronto Rehabilitation Institute, University Health Network, 550 University Avenue, Toronto, ON M5G 2A2 Canada; 5 Queen’s University, 99 University Avenue, Kingston, ON K7L 3N6 Canada; 6 Present Address: Department of Psychiatry, Temerty Faculty of Medicine, University of Toronto, 250 College Street, Toronto, ON M5T 1R8 Canada

**Keywords:** Slow-paced breathing, Cognition, Cognitive decline, Aging, Neurorehabilitation, Paced breathing, Heart rate variability, Executive function, Memory, Attention

## Abstract

**Background:**

Slow-paced breathing (SPB) has emerged as an intervention to improve cognitive function and prevent cognitive decline. The proposed systematic review aims to consolidate previous literature examining the impacts of SPB across cognitive subdomains when compared to passive and active controls in adults, as well as the mechanisms involved.

**Methods:**

Literature searches will be conducted in MEDLINE(R) ALL, Embase Classic + Embase, Cochrane Central Register of Controlled Trials, PsycINFO, CINAHL Ultimate, and Web of Science. Gray literature sources include preprints and clinical trial registries. Citation tracking will be used as a supplemental search method. RCTs, quasi-randomized trials, and non-randomized interventions with a control or comparison group focused on adult human participants will be included. Studies on pediatric populations or animals will be excluded. The primary outcomes are standardized cognitive test scores and test batteries. Data on the parameters of SPB protocols will also be collected to gain insight into the mechanisms driving observed cognitive changes. Two independent reviewers will blindly complete citation screening, data extraction, risk-of-bias assessment using Covidence, and appraisal of study quality using GRADEpro. Risk of bias will be assessed for non-randomized controlled trials and RCTs using the ROBINS-I and RoB tools, respectively. A narrative synthesis of the findings will be conducted. Results will be stratified based on methodological features such as study design, population demographics, intervention parameters, and cognitive domain. If the data of the included literature permit, a meta-analysis will be performed to compare improvements by (i) cognitive domain, (ii) population demographics, and (iii) parameters of intervention protocols (e.g., duration of inhalation vs. exhalation).

**Discussion:**

SPB is a promising intervention for conferring generalizable cognitive improvements. This review will consolidate early findings, give insight into SPB’s efficacy as a clinical intervention, and lay the groundwork for future research into mechanisms and optimal parameters.

**Systematic review registration:**

PROSPERO CRD42024615253

**Supplementary Information:**

The online version contains supplementary material available at 10.1186/s13643-025-03004-w.

## Background

Slow-paced breathing (SPB) refers to the controlled process whereby an individual inhales and exhales at a slower pace [1]. SPB is defined as taking less than 10 breaths per minute [2], compared to the average adult taking 12–20 breaths per minute [3]. Controlling the breath in this way plays a direct role in the measured heart rate variability (HRV) or variation in time between heart beats, with a higher HRV indicating better adaptive mechanisms within the autonomic nervous system [4]. Owing to the overlapping neural circuits involved in both cognition and the cardiac autonomic system, HRV status has been associated with cortical activity and cognition [5]. Several studies have shown evidence of these relationships, including (i) correlations between cardiac vagal activity (or vagal tone) and executive functioning (including cognitive flexibility) [6], and the autonomic stress response [7], and (ii) the effect of SPB on HRV, which influences neuroplasticity and higher-order cognitive functions [8–10].

Studies of SPB interventions in both healthy adults and clinical populations have found significant improvements in attention and executive functions, including attentional focus, decision-making, task switching, and working memory [2, 5, 9, 11–15]. One study revealed a correlation between increased cardiac vagal activity and parasympathetic activity after SPB, and increased scores in stimulus-response learning and adaptive relearning [8]. Another study found that resonance breathing led to increased parasympathetic and decreased sympathetic activity, as well as improvements in visual‒motor speed and attention [16]. For this reason, SPB shows promise in improving cognition in both healthy individuals and potentially in neurological populations, where cognitive decline is more pronounced and accelerated [16–19]. Cognitive decline does not just affect neurological populations. For instance, in a study of 2431 American adults over the age of 65, 40.1% of individuals were classified as having early-stage memory impairment, 6.6% were classified as having late-stage memory impairment, and the weighted prevalence of memory impairment was 47% [20]. Moreover, changes in the brain and cognition are not limited to older adult populations. An analysis of 56 MRI studies with a total of 2211 participants observed that after age 35, brain volume begins to steadily decrease [21]. Additionally, fluid intelligence, the ability to solve unfamiliar problems via inductive and deductive reasoning, is shown to peak during the mid-20s before beginning to decline [22–24]. Finally, traumatic brain injuries, which occur in young people more frequently than adults [25], can cause significant cognitive impairments.

Accessible, cost-effective interventions that demonstrate efficacy and generalizability are critically needed to address these cognitive concerns. In this context, SPB is a promising candidate due to its scalability, barrier-free access, and efficacy demonstrated in prior studies [2, 5, 9, 11–15].

To date, there has been extensive research examining the impact of SPB on emotional functioning, e.g., stress and anxiety levels, and physical health, including cardiovascular functioning, heart rate variability, and blood pressure [26–29], and this substantial research is reflected in the many available systematic reviews on these topics [2, 11, 30]. However, there has been considerably less research focusing primarily on SPB and its impact on cognition. Additionally, this literature on the impact of SPB on cognition has yet to be consolidated in a systematic review, and many questions remain unanswered. Our systematic review aims to address these gaps. Therefore, the goal of this systematic review is to consolidate previous findings across studies that explore the effect of SPB on cognition, the putative driving mechanisms behind these effects, and the optimal intervention parameters employed in SPB studies.

Previous literature has explored individual cognitive domains using SPB interventions of varying durations and frequencies. However, the tests used to measure cognitive outcomes are inconsistent between studies making it difficult to compare findings and understand the overall effect of SPB, as well as the optimal duration and frequency needed to see significant cognitive improvement. In order to better understand the effects of SPB on cognition and support its implementation as a clinical intervention for cognitive improvements, a systematic review is needed to consolidate and highlight significant findings. The review will address the following questions:

(i) In healthy populations, does SPB lead to cognitive change (domain-specific or more generally)?
(ii) What parameters of SPB drive cognitive change and how are these parameters mechanistically linked?

## Methods/Design

### Guidelines and registration

This review protocol is informed by the Cochrane Handbook for Systematic Reviews of Interventions and the Preferred Reporting Items for Systematic review and Meta-analysis Protocols (PRISMA-P) 2015 [31, 32]. Our eventual review will be informed by analogous guidelines for systematic reviews. Our review is registered with the International Prospective Register of Systematic Reviews (PROSPERO CRD42024615253). Any modifications to our protocol will be formally recorded as amendments in PROSPERO.

### Eligibility criteria

No restrictions will be placed on language or publication date during screening. Articles written in any language other than English will be translated using the Google Translate or DeepL AI platforms. The included studies will meet the Participants, Interventions, Comparator, Outcome, and Type of study (PICOT) guidelines below [33, 34].

#### Participants

We will include only studies involving healthy, adult human participants aged 18+ years. More specifically, studies involving pediatric populations or animal models will be excluded.

#### Intervention

Our systematic review will focus on only randomized controlled trials, quasi-randomized controlled trials, and non-randomized studies of interventions with a control or comparator group; that is, studies must include a comparator group (whether created through random allocation or otherwise) against which an SPB intervention is compared. Eligible studies must examine the effect of breathing interventions alone; studies examining the impact of SPB in combination with other interventions, such as exercise, meditation, cognitive training, and yoga, will not be included owing to potentially confounding effects. Eligible studies must test the effect of SPB or breath-pacing interventions on participants’ ability to perform cognitive tasks. SPB encompasses a variety of breathing techniques and does not have a singular definition. SPB also has a wide range of alternate names. For our review, any intervention that includes SPB, regardless of terminology, will be included (for example, slow-paced breathing/respiration, resonance breathing and resonant frequency breathing, square breathing, box breathing, 4-4-8 or 4–7-8 breathing, pranayama, nadi shodhana, unilateral nostril breathing, and yoga breathing). Please see Additional File 1 for a comprehensive list of intervention search terms. Studies examining relaxation techniques will be included only if the technique is SPB or deep breathing.

#### Comparators

Any study with an active or passive control will be included. An active control may include another type of intervention (e.g., yoga, stretching, exercise, education), including another breathing intervention. Passive control conditions generally refer to a lack of intervention or a waitlist condition.

#### Outcomes

The primary outcome is cognitive test performance. Eligible studies may have single or multiple outcome measures for cognition. We will include studies looking at changes in the following cognitive domains: executive functioning (including working memory, inhibitory control, error monitoring, and decision-making), verbal and visuospatial attention (both timed and untimed), learning and memory (both verbal and visuospatial, and short delay and long delays), and visual motor speed and dexterity. These cognitive domains will be measured via improvements in accuracy or speed on cognitive batteries (e.g., the mini-mental state examination [35], Montreal Cognitive Assessment [36]) and discrete cognitive tests and subtests [37, 38]. Qualifying studies will include outcome measures taken immediately after the SPB intervention and/or after a period of time has passed (either since performing the SPB intervention or consistently performing the SPB intervention).

The secondary outcomes (not included in the primary search criteria) are physiological mechanisms, such as heart rate and heart rate variability. For studies included in the final review, we will conduct a supplementary post-screening to identify these secondary outcomes and to identify proposed mechanisms by which SPB may influence cognition. This analysis of secondary outcomes will be narrative and based on the results of the primary search.

#### Type of study

##### Inclusion criteria

Qualifying studies must be one of the following — randomized controlled trials (RCTs) and controlled clinical trials (CCTs), including quasi-randomized controlled trials and non-randomized trials with a comparison or control group.

##### Exclusion criteria

Review articles, observational studies, and conference abstracts/proceedings will not be included.

### Information sources

A comprehensive search strategy was developed in consultation with an information specialist (C. C.). The search will be conducted across the following electronic databases: MEDLINE(R) ALL (Ovid), Embase Classic + Embase (Ovid), Cochrane Central Register of Controlled Trials (Ovid), PsycInfo (Ovid), CINAHL Ultimate (EBSCO), and Web of Science (Clarivate). Gray literature sources include preprints found in the electronic databases and a search of clinical trial registries (ClinicalTrials.gov and the International Clinical Trials Registry Platform) for unpublished results. Citation tracking of included studies will be performed to find additional articles that meet the eligibility criteria. The initial search will be completed in May 2025 and will cover studies in the databases from inception. Prior to publication of the systematic review, the search will be updated to capture any relevant studies published since the last search date.

### Search strategy

The initial search strategy was developed in MEDLINE(R) ALL (Ovid) and tested using a set of target articles provided by the team. The search strategy included a combination of subject headings (i.e., Medical Subject Headings, MeSH) and keywords. Truncation, adjacency operators, and appropriate use of the Boolean operators (OR and AND) were incorporated in the search strategy to ensure a comprehensive search of the literature.

The search strategy was organized into two search concepts: (slow-paced breathing) AND (randomized controlled trials/controlled clinical trials). Some search terms for SPB included the following: slow-paced breathing, slow breathing, coherent breathing, resonance breathing, and pranayama. A sensitive filter developed by Canada’s Drug Agency (formerly Canadian Agency for Drugs and Technologies in Health) was used to search for RCTs and CCTs, including quasi-randomized controlled trials and non-randomized trials with a comparison or control group [39]. Studies on animals and children will be excluded from the search strategy by applying a filter if one is available in the databases. An exclusion filter for studies involving animals or children is not available for Web of Science, ClinicalTrials.gov, and ICTRP. No limits were applied for language or publication date.

A sample of 200 articles from the initial MEDLINE search was screened to identify additional search terms and make revisions to the search, using an iterative approach involving feedback from the team and suggestions from the information specialist to improve the search. In addition, the MEDLINE search was peer reviewed by another information specialist using the Peer Review of Electronic Search Strategies (PRESS) form. We thank Emilia Main, MI (Toronto Rehabilitation Institute, UHN), for peer review of the submitted search strategy. The final MEDLINE search strategy can be found in Additional File 1 and will be translated to the other electronic databases, using the appropriate syntax and subject headings. In Cochrane Central Register of Controlled Trials, the RCT/CCT filter will not be applied since the database only contains reports of randomized and quasi-randomized controlled trials.

In addition, a search of the gray literature will be conducted in ClinicalTrials.gov and the International Clinical Trials Registry Platform (ICTRP) to find unpublished results. Other gray literature sources, such as preprints, will also be included in the search strategy of the electronic databases. After screening has been completed, forward and backward citation tracking of the included studies will be used as a supplemental search method.

### Data management

The database search results will be exported as .ris files and imported into Covidence, which will be used for de-duplication, title and abstract screening, full-text screening, and data extraction. Zotero will be used to manage citations.

Note that search results pertaining to secondary outcomes (i.e., physiological mechanisms) will also be imported into Covidence and contained in a second and separate file from the primary search results.

### Screening and selection process

All the citations returned by our search will be independently screened by two blinded reviewers (S. R. and C. T.) in Covidence. Before screening the official list of citations returned from the database searches, both reviewers will complete a pilot screening of a subset of citations (i.e., a training set) to determine inter-rater reliability and refine the eligibility criteria based on the PICOT criteria (i.e., Participants, Intervention, Comparator, Outcomes, Type of study), referred to above. After completing the practice set, the two independent reviewers will proceed to the blinded formal screening by examining the title and abstract of the articles to determine eligibility. The full texts of the qualifying articles will then be screened by both reviewers to further narrow down the citations based on the eligibility criteria. Discrepancies will be resolved by discussion with a third reviewer to decide whether to include the article. Randomized controlled trials, quasi-randomized controlled trials, and non-randomized studies of interventions with a control or comparator group will be included, while observational studies will be excluded to maintain a high standard of clinical relevance and to homogenize the data for simplified analysis. A PRISMA flow diagram will be used to summarize the search results and the study selection process [31].

### Data extraction

The data extraction form will be designed and completed in Covidence for each of the eligible studies. In studies where important data are missing (e.g., breathing protocol parameters), we will attempt to contact the authors of the paper to obtain the missing information. If this is unsuccessful and we cannot acquire the missing data, we will state the information as “not reported” in our data extraction table.

All breathing intervention parameters reported in each study will be extracted. To facilitate comparison across studies, we will standardize and record intervention characteristics. For example, based on the final review results, we will likely report intervention duration (in weeks), frequency (days per week), and session length (in minutes). The breathing protocol will be recorded in detail, including the presence or absence of breath holds and the duration of each breath component (e.g., inhale-hold-exhale-hold in seconds). Additional parameters such as breathing frequency (breaths per minute) and the type of SPB intervention (e.g., belly breathing, unilateral nostril breathing) will also be extracted.

For outcome measures, we will extract pre- and post-intervention scores, the cognitive test(s) used, the domain(s) assessed (e.g., memory, attention), and any reported effect estimates (e.g., mean differences, standardized mean differences), confidence intervals, and *p*-values. These outcome measures will be analyzed to determine the impact of slow-paced breathing on cognition, and the effect estimates will allow us to calculate the overall effect size for the meta-analysis. Confidence intervals and *p*-values will help assess the precision and significance of the findings. This detailed extraction will support consistent standardization across studies and enable both narrative synthesis and potential meta-analysis.

Data extraction will be performed independently and in a blinded manner by two team members (S. R. and C. T.). To ensure consistency, a third team member (B. S.) will compare the completed forms for discrepancies. Any discrepancies will be resolved by referencing the original article. Before full data extraction, the reviewers will pilot the process on a sample of studies to ensure consistency and accuracy.

### Assessment of study quality

To determine the risk of bias in the studies included in our review after screening, nonrandomized controlled trials and non-randomized interventions with a control or comparison group will be assessed via the Risk of Bias in Non-Randomized Studies - of Interventions (ROBINS-I) risk-of-bias tool [40], and RCTs will be assessed via the Risk of Bias (RoB) tool [41].

Seven bias domains are explored in the ROBINS-I risk-of-bias tool [40]. This includes bias due to confounding factors, bias in the selection of participants for the study, bias in the classification of interventions, bias due to deviations from intended interventions, bias due to missing data, bias in the measurement and analysis of outcomes, and bias in the selection of the reported result. Five domains are explored in the RoB tool [41]. This includes bias due to deviations from intended interventions, bias due to missing outcome data, bias in the measurement of the outcome, and bias in the selection of the reported result.

The appropriate risk-of-bias assessment for each study will be conducted independently by two blinded reviewers (S. R. and C. T.) in Covidence. Discrepancies will be resolved by a third reviewer (B. S.). Studies rated as having a high risk of bias will be included in the review but clearly identified, and their findings will be interpreted with caution to ensure transparency about the quality of the evidence. Inter-rater reliability will be assessed by having all reviewers individually assess a subset of included studies and represented using Cohen’s kappa statistic, which will be calculated by a third reviewer (B. S.) [42]. Disagreements will be discussed between reviewers or with an additional reviewer if needed. If the Cohen’s kappa value is below 0.60, the rating criteria will be revisited.

### Data synthesis

Given the considerable heterogeneity observed across the included studies, we anticipate conducting a structured narrative synthesis to comprehensively analyze the findings, following the Synthesis Without Meta-analysis (SWiM) checklist [43]. The SWiM checklist will complement and be used as an extension to PRISMA [43]. This synthesis will consider stratifying results based on methodological features. This may include study design (e.g., prospective vs. retrospective, randomized vs non-randomized) and population demographics (e.g., age, sex, gender). It may also include features of the intervention, including parameters of SPB (e.g., duration of each session, duration of overall intervention, breathing frequency, equal vs differing inhalation vs. exhalation, nostril manipulation [presence/absence and type], and belly breathing [presence/nature]), and outcome measures of the SPB intervention, namely the cognitive tests and domains, as well as physiological outcomes where available (e.g., heart rate variability). These stratifications will enable us to draw meaningful comparisons, identify trends or patterns across studies, and report potential limitations in the findings, as per the SWiM checklist [43].

Where possible, we will complement the narrative synthesis with a meta-analysis. Eligibility for meta-analysis will be determined based on both conceptual similarity using the PICOT framework and statistical heterogeneity (*I*^2^). Potential subgroups for meta-analysis will be identified based on these elements, for instance, grouping studies by population demographics (e.g., age, sex, gender), intervention characteristics (e.g., protocol length, SPB technique such as unilateral nostril breathing or belly breathing), comparator type, and cognitive outcome domain. This will depend on the availability of sufficient and comparable data related to cognitive outcomes. For the meta-analysis, cognitive outcomes will be grouped based on shared domains (e.g., memory, attention, executive function) or specific common cognitive tests employed across studies. In addition to the primary analysis, we will address specific secondary questions to further explore the efficacy of the interventions. These questions will consider moderators of outcomes, specifically how breathing duration and breathing patterns (e.g., equal inhalation-to-exhalation ratios versus differing ratios) affect cognitive outcomes. By analyzing these moderators, we aim to provide a more comprehensive understanding of the factors that influence the effectiveness of the interventions.

#### Meta-analysis model

We will use a random-effects meta-analysis model, as we expect true effect sizes to vary across studies due to differences in interventions (e.g., unilateral nostril breathing, belly breathing), cognitive domain outcomes (e.g., attention, memory), and methods (e.g., different measurement techniques) in healthy populations [44]. The model will pool effect size types (e.g., standardized mean differences) using inverse-variance weighting and estimate between-study variance (*τ*^2^) using the DerSimonian and Laird or REML approach [44].

Standardized mean differences (SMDs) will serve as the metric to facilitate the comparison of results across studies that may utilize different cognitive assessment tools. Additionally, mean differences will be calculated to demonstrate changes in cognitive test scores from pre- to post-intervention, providing insight into the efficacy of SPB interventions. Suitability of the included studies for a meta-analysis will be assessed using the *I*^2^ statistic [45]. Heterogeneity represented by results under 40% will be considered negligible and suitable for a meta-analysis. Results over 40% will be represented by a narrative synthesis, which will be conducted based on the data available in the included studies in one of three ways. If available, the median, interquartile range, and range will be used to summarize effect estimates. If only *p*-values and direction of effect are available, authors will combine *p*-values. If only the direction of effect is available, authors will do vote counting based on the direction.

The data to be synthesized will be extracted systematically from each included study, following the criteria outlined in the “Data Extraction” section above. These extracted data points will encompass detailed information on participant demographics (e.g., age, sex), the specifics of the SPB intervention (e.g., frequency of breaths per minute, relative duration of inhalation vs. exhalation, session length, and intervention duration), and the outcomes assessed (e.g., types of cognitive tests, physiological measures).

### Confidence in cumulative evidence

After analysis has been completed, the Grading of Recommendations, Assessment, Development, and Evaluation (GRADE) approach will be used, if applicable, to assess the certainty of evidence for each cognitive outcome investigated across studies by evaluating the overall body of evidence for each outcome separately based on the following criteria: risk of bias, inconsistency, indirectness, imprecision, and publication bias [32]. This evaluation’s result will determine the evidence certainty levels as being high, moderate, low, or very low [32]. For transparency, all findings will be presented in a summary of findings table for each of the cognitive domains analyzed. The software GRADEpro will be used [46]. Two blinded reviewers (S. R. and C. T.) will independently complete the table; any discrepancies will be resolved by a third reviewer (B. S.). Studies rated as having low or very low quality evidence will still be included in the review, but clearly identified, and their findings will be interpreted with caution to ensure transparency about the quality of the evidence.

## Discussion

Several interventions have been developed to impact cognitive function and aging-related cognitive decline in healthy adults. Among these, commercial "brain games" are some of the most popular and notable. Unfortunately, there is limited evidence to suggest that these interventions lead to improvements in cognitive abilities beyond the specific tasks on which participants are trained [47, 48]. That is, there is a dearth of evidence regarding the generalizability (aka “transfer”) of benefits to everyday life, whereby participants improve in their cognitive capacities on outcomes beyond those on which they were trained, with evidence of improved cognitive capacities in daily functioning [49]. In the context of clinical populations, the efficacy of interventions designed to promote generalizable cognitive recovery after brain insult, mitigate decline in the context of Mild Cognitive Impairment, or slow the onset of dementia has also been only modest to date [50]. Most interventions show improvements in the skills trained (e.g., on the attention tests used during training). However, many studies state that these interventions have modest generalizability (or the absence of measured generalizeability) to everyday life [51], with very few studies reporting on the acquired strategies being used by participants in everyday life [52].

In contrast, in recent years, SPB has emerged as a promising modality for enhancing or protecting cognitive function, one that is non-invasive, accessible, and easy to implement. By consolidating the literature on SPB and cognition, this systematic review will further elucidate this area of treatment research and will help to generate novel hypotheses for future research. The scale of cognitive decline, the multiple etiologies, and wide-ranging barriers to treatment (e.g., costs, geography) make treatment for cognition a considerable challenge. Self-administered treatments (without the costs of a therapist) and minimal-risk approaches with the potential to confer benefits regardless of etiology are needed. SPB has shown efficacy for improving not only physiological health (including aerobic endurance, and sleep quality and diastolic blood pressure) [30] and mental health (including depression and anxiety symptoms) [2] but also cognition [11–13, 53].

Thus, SPB stands to be an efficacious, accessible, and noninvasive way to improve cognitive function on a vast scale, as it does not necessitate a therapist or special equipment. Future developments may thus include self-administration of the intervention, which would increase accessibility and eliminate costs. As a self-administered or a guided treatment, it can be administered remotely. Importantly, SPB is a practice that can be implemented as a preventative measure. Cognition declines with age, and SPB has shown strong evidence for improving cognition in older populations [8], potentially helping to buffer against aging or disease-related cognitive decline. Therefore, the findings in this review will have a significant impact on future research efforts to mitigate cognitive decline in both healthy adults and neurological populations.

## Supplementary Information

Additional file 1. Medline Search Strategy. This search strategy outlines the parameters used to produce the selection of studies outlined by the PICOT framework.

Additional file 2. PRISMA-P 2015 Checklist.

Additional file 3. PRISMA 2020 for Abstracts Checklist.

## Data Availability

The authors confirm that the data supporting the review findings of this study will be available within the article and/or its supplementary materials.

## Abbreviations

SPB: Slow-paced breathing
HRV: Heart rate variability
RCT: Randomized controlled trial
CCT: Controlled clinical trial
PRISMA: Preferred Reporting Items for Systematic reviews and Meta-Analyses
PROSPERO: International Prospective Register of Systematic Reviews
