# Supplementary material for: A systematic review protocol for slow-paced breathing in healthy populations: Impacts on cognition and insights into mechanisms of action
Source: Syst Rev. 2025 Dec 7;15:6. doi: 10.1186/s13643-025-03004-w (PMC12797364; doi:10.1186/s13643-025-03004-w)
Supplement: Supplementary file 1 — Additional file 1. Medline Search Strategy. This search strategy outlines the parameters used to produce the selection of studies outlined by the PICOT framework. [file 13643_2025_3004_MOESM1_ESM.docx]

Medline Search Strategy

**Database:** Ovid MEDLINE(R) ALL <1946 to October 30, 2025>

| **#** | **Query** | **Results from 31 Oct 2025** |
| --- | --- | --- |
| 1 | Breathing Exercises/ | 4,347 |
| 2 | ((slow-pace? or slowpace?) adj3 (breath* or respir* or inspir* or inhal* or expir* or exhal*)).tw,kf. | 125 |
| 3 | (slow* adj1 (breath* or respire* or respiring or respiration*)).tw,kf. | 604 |
| 4 | (slow* down adj2 (breath* or respire* or respiring or respiration*)).tw,kf. | 34 |
| 5 | (paced adj3 (breath* or respir* or inspir* or inhal* or expir* or exhal*)).tw,kf. | 634 |
| 6 | (pacing adj (breath* or respir* or inspir* or inhal* or expir* or exhal*)).tw,kf. | 12 |
| 7 | breath control.tw,kf. | 152 |
| 8 | (controlled rate? adj3 (breath* or respir* or inspir* or inhal* or expir* or exhal*)).tw,kf. | 2 |
| 9 | (controlled adj3 breathing rate*).tw. | 20 |
| 10 | controlled breathing rate*.kf. | 0 |
| 11 | ((resonant or resonance frequenc*) adj3 (breath* or respir* or inspir* or inhal* or expir* or exhal*)).tw,kf. | 105 |
| 12 | (resonance adj (breath* or respir* or inspir* or inhal* or expir* or exhal*)).tw,kf. | 32 |
| 13 | (resonance adj3 breathing rate*).tw,kf. | 2 |
| 14 | (coherent adj3 (breath* or respir* or inspir* or inhal* or expir* or exhal*)).tw,kf. | 65 |
| 15 | (metronom* adj3 (breath* or respir* or inspir* or inhal* or expir* or exhal*)).tw,kf. | 106 |
| 16 | ((slow* or paced or pacing or controlled or deep* or regulated or measured) adj3 (breathing exercise* or breathing technique* or breathing intervention* or breathing protocol* or breathing training*)).tw. | 447 |
| 17 | ((slow* or paced or pacing or controlled or deep* or regulated or measured) adj (breathing exercise* or breathing technique* or breathing intervention* or breathing protocol* or breathing training*)).kf. | 24 |
| 18 | (volitional* adj2 (breath* or respir* or inspir* or inhal* or expir* or exhal*)).tw,kf. | 93 |
| 19 | (fixed adj (breath* or respir* or inspir* or inhal* or expir* or exhal*)).tw,kf. | 140 |
| 20 | (structured adj (breath* or respir* or inspir* or inhal* or expir* or exhal*)).tw,kf. | 30 |
| 21 | (timed adj (breath* or respir* or inspir* or inhal* or expir* or exhal*)).tw,kf. | 45 |
| 22 | ((diaphragm* or abdominal or abdomen or belly) adj (breath* or respire* or respiring or respiration*)).tw,kf. | 876 |
| 23 | (relaxed breathing or calm breathing or roll breathing).tw,kf. | 73 |
| 24 | ((vagal or vagus) adj2 (breath* or respir* or inspir* or inhal* or expir* or exhal*)).tw,kf. | 193 |
| 25 | (pursed lip? adj3 (breath* or respir* or inspir* or inhal* or expir* or exhal*)).tw,kf. | 194 |
| 26 | ((alternate nostril? or uninostril or uni-nostril or unilateral nostril? or right nostril? or left nostril?) adj3 (breath* or respir* or inspir* or inhal* or expir* or exhal*)).tw,kf. | 128 |
| 27 | ((alternate or unilateral) adj2 (nostril adj (breath* or respir* or inspir* or inhal* or expir* or exhal*))).tw,kf. | 94 |
| 28 | slow nasal breath*.tw,kf. | 2 |
| 29 | (deep* adj1 (breath* or respire* or respiring or respiration*)).ti,kf. | 514 |
| 30 | (slow adj3 deep adj3 breath*).tw,kf. | 201 |
| 31 | (slow exhalation* or extended exhalation*).tw,kf. | 32 |
| 32 | ((yoga or yogic) adj2 (breath* or respir* or inspir* or inhal* or expir* or exhal*)).tw,kf. | 452 |
| 33 | ((square or box or boxed) adj (breath* or respir* or inspir* or inhal* or expir* or exhal*)).tw,kf. | 33 |
| 34 | (cadence adj3 (breath* or respir* or inspir* or inhal* or expir* or exhal*)).tw,kf. | 21 |
| 35 | regulated breath*.tw,kf. | 53 |
| 36 | (tactical* adj2 (breath* or respire* or respiring or respiration*)).tw,kf. | 4 |
| 37 | (cyclic* adj2 (breath* or respire* or respiring or respiration*)).tw,kf. | 96 |
| 38 | ((4-4-8 or 4-7-8) adj3 breath*).tw,kf. | 7 |
| 39 | guided breath*.tw,kf. | 145 |
| 40 | (pranayam* or kumbhak or kumbhaka or puraka or rechaka or nadi shodhan* or nadishodhan* or anulom vilom).tw,kf. | 621 |
| 41 | (sudarshan kriya or rhythmic breath*).tw,kf. | 226 |
| 42 | (ujjayi or ocean breath* or victorious breath* or conquering breath*).tw,kf. | 14 |
| 43 | (bhramari or bee breath*).tw,kf. | 47 |
| 44 | or/1-43 [Slow Paced Breathing Concept] | 8,871 |
| 45 | (Randomized Controlled Trial or Controlled Clinical Trial or Pragmatic Clinical Trial or Equivalence Trial or Clinical Trial, Phase III).pt. | 745,403 |
| 46 | Randomized Controlled Trial/ | 648,599 |
| 47 | exp Randomized Controlled Trials as Topic/ | 192,287 |
| 48 | "Randomized Controlled Trial (topic)"/ | 0 |
| 49 | Controlled Clinical Trial/ | 95,746 |
| 50 | exp Controlled Clinical Trials as Topic/ | 198,216 |
| 51 | "Controlled Clinical Trial (topic)"/ | 0 |
| 52 | Randomization/ | 108,984 |
| 53 | Random Allocation/ | 108,984 |
| 54 | Double-Blind Method/ | 186,496 |
| 55 | Double Blind Procedure/ | 0 |
| 56 | Double-Blind Studies/ | 186,496 |
| 57 | Single-Blind Method/ | 35,680 |
| 58 | Single Blind Procedure/ | 0 |
| 59 | Single-Blind Studies/ | 35,680 |
| 60 | Placebos/ | 36,154 |
| 61 | Placebo/ | 0 |
| 62 | Control Groups/ | 2,153 |
| 63 | Control Group/ | 2,153 |
| 64 | (random* or sham or placebo*).ti,ab,hw,kf. | 2,085,978 |
| 65 | ((singl* or doubl*) adj (blind* or dumm* or mask*)).ti,ab,hw,kf. | 289,922 |
| 66 | ((tripl* or trebl*) adj (blind* or dumm* or mask*)).ti,ab,hw,kf. | 2,241 |
| 67 | (control* adj3 (study or studies or trial* or group*)).ti,ab,kf. | 1,430,001 |
| 68 | (Nonrandom* or non random* or non-random* or quasi-random* or quasirandom*).ti,ab,hw,kf. | 63,898 |
| 69 | allocated.ti,ab,hw. | 98,061 |
| 70 | ((open label or open-label) adj5 (study or studies or trial*)).ti,ab,hw,kf. | 53,315 |
| 71 | ((equivalence or superiority or non-inferiority or noninferiority) adj3 (study or studies or trial*)).ti,ab,hw,kf. | 15,850 |
| 72 | (pragmatic study or pragmatic studies).ti,ab,hw,kf. | 738 |
| 73 | ((pragmatic or practical) adj3 trial*).ti,ab,hw,kf. | 9,911 |
| 74 | ((quasiexperimental or quasi-experimental) adj3 (study or studies or trial*)).ti,ab,hw,kf. | 16,917 |
| 75 | (phase adj3 (III or "3") adj3 (study or studies or trial*)).ti,hw,kf. | 41,045 |
| 76 | or/45-75 [CADTH RCT/CCT Filter for Medline/Embase] | 2,977,149 |
| 77 | 44 and 76 | 3,113 |
| 78 | 77 not (exp animals/ not humans.sh.) | 3,073 |
| 79 | 78 not ((exp infant/ or exp child/ or adolescent/) not exp adult/) | 2,912 |
