# Supplementary material for: A systematic review protocol for slow-paced breathing in healthy populations: Impacts on cognition and insights into mechanisms of action
Source: Syst Rev. 2025 Dec 7;15:6. doi: 10.1186/s13643-025-03004-w (PMC12797364; doi:10.1186/s13643-025-03004-w)
Supplement: Supplementary file 3 — Additional file 3. PRISMA 2020 for Abstracts Checklist. [file 13643_2025_3004_MOESM3_ESM.docx]

**PRISMA 2020 for Abstracts Checklist**

| **Section and Topic** | **Item #** | **Checklist item** | **Reported (Yes/No)** | **Line Number** |
| --- | --- | --- | --- | --- |
| **TITLE** | | | | |
| Title | 1 | Identify the report as a systematic review. | Yes | 2-3 |
| **BACKGROUND** | | | | |
| Objectives | 2 | Provide an explicit statement of the main objective(s) or question(s) the review addresses. | Yes | 24-26 |
| **METHODS** | | | | |
| Eligibility criteria | 3 | Specify the inclusion and exclusion criteria for the review. | Yes | 31-33 |
| Information sources | 4 | Specify the information sources (e.g. databases, registers) used to identify studies and the date when each was last searched. | Yes  (However, we did not include the date of last search as searches are still in progress) | 28-30 |
| Risk of bias | 5 | Specify the methods used to assess risk of bias in the included studies. | Yes | 38-40 |
| Synthesis of results | 6 | Specify the methods used to present and synthesise results. | Yes | 40-45 |
| **RESULTS** | | | | |
| Included studies | 7 | Give the total number of included studies and participants and summarise relevant characteristics of studies. | N/A |  |
| Synthesis of results | 8 | Present results for main outcomes, preferably indicating the number of included studies and participants for each. If meta-analysis was done, report the summary estimate and confidence/credible interval. If comparing groups, indicate the direction of the effect (i.e. which group is favoured). | N/A |  |
| **DISCUSSION** | | | | |
| Limitations of evidence | 9 | Provide a brief summary of the limitations of the evidence included in the review (e.g. study risk of bias, inconsistency and imprecision). | N/A |  |
| Interpretation | 10 | Provide a general interpretation of the results and important implications. | N/A |  |
| **OTHER** | | | | |
| Funding | 11 | Specify the primary source of funding for the review. | Yes | 53-54 |
| Registration | 12 | Provide the register name and registration number. | Yes | 50 |

*From:* Page MJ, McKenzie JE, Bossuyt PM, Boutron I, Hoffmann TC, Mulrow CD, et al. The PRISMA 2020 statement: an updated guideline for reporting systematic reviews. BMJ 2021;372:n71. doi: 10.1136/bmj.n71
